# Supplementary figures and images for: Rapid and reproducible haplotyping of complete mitochondrial genomes using split k-mers
Source: BMC Genomics. 2026 Apr 11;27:477. doi: 10.1186/s12864-026-12811-x (PMC13181888; doi:10.1186/s12864-026-12811-x)

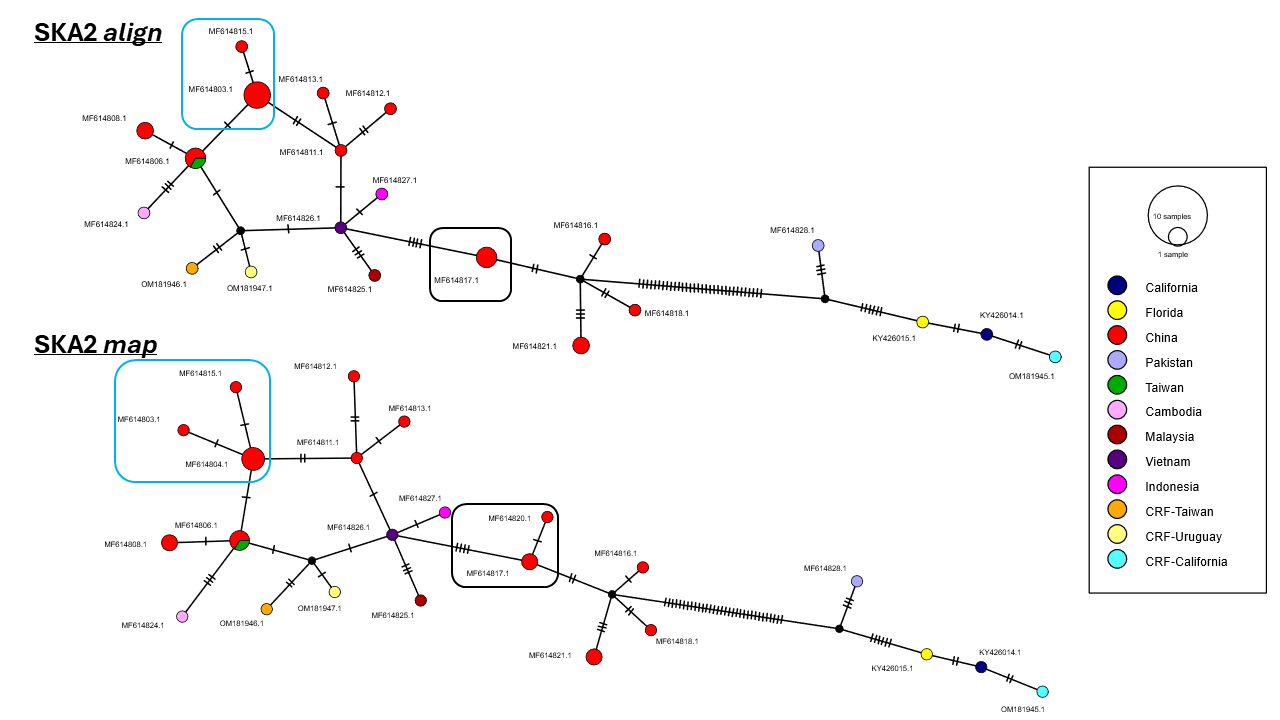

Supplement: Supplementary file 1 — Supplementary Material 1. [file 12864_2026_12811_MOESM1_ESM.tiff]

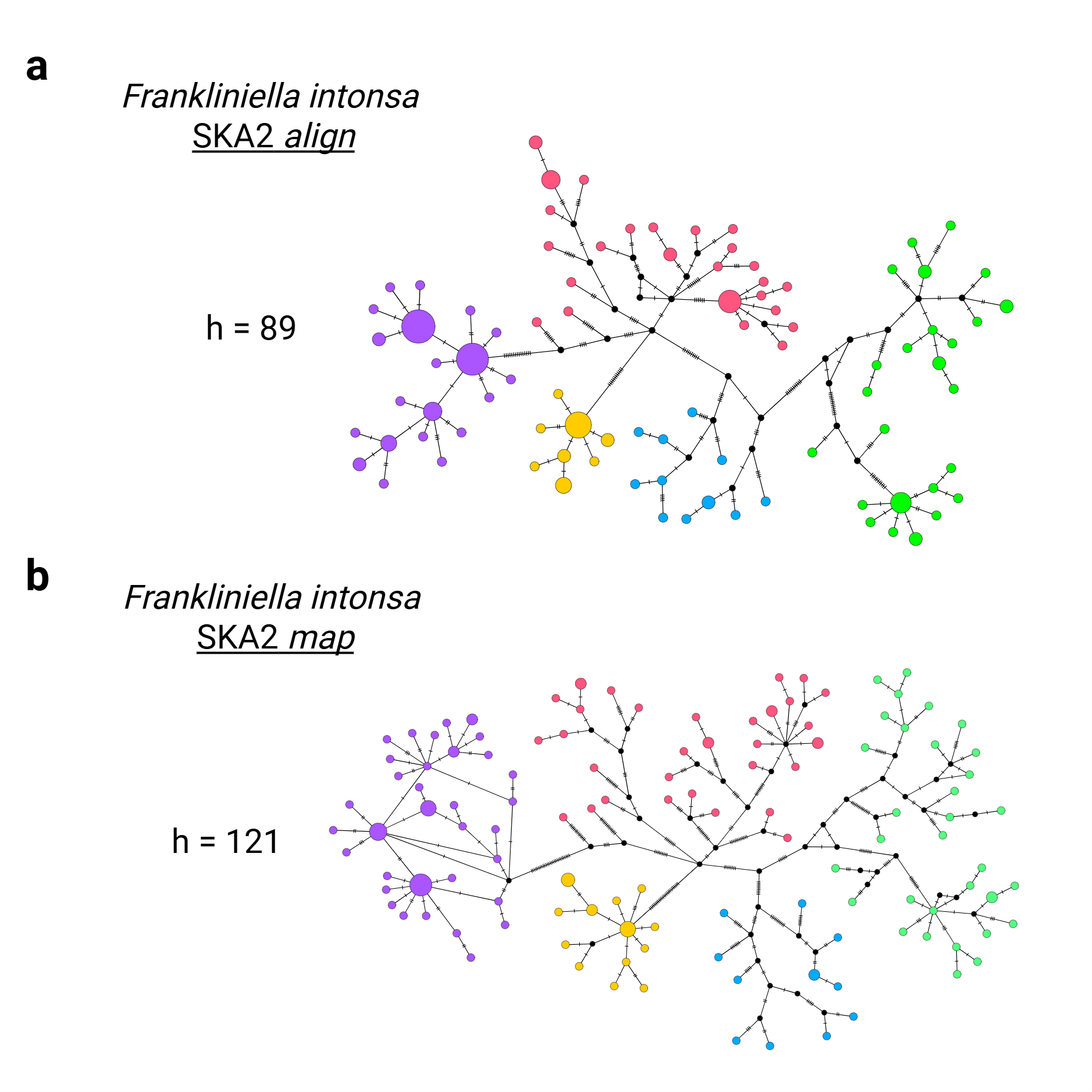

Supplement: Supplementary file 2 — Supplementary Material 2. [file 12864_2026_12811_MOESM2_ESM.tiff]

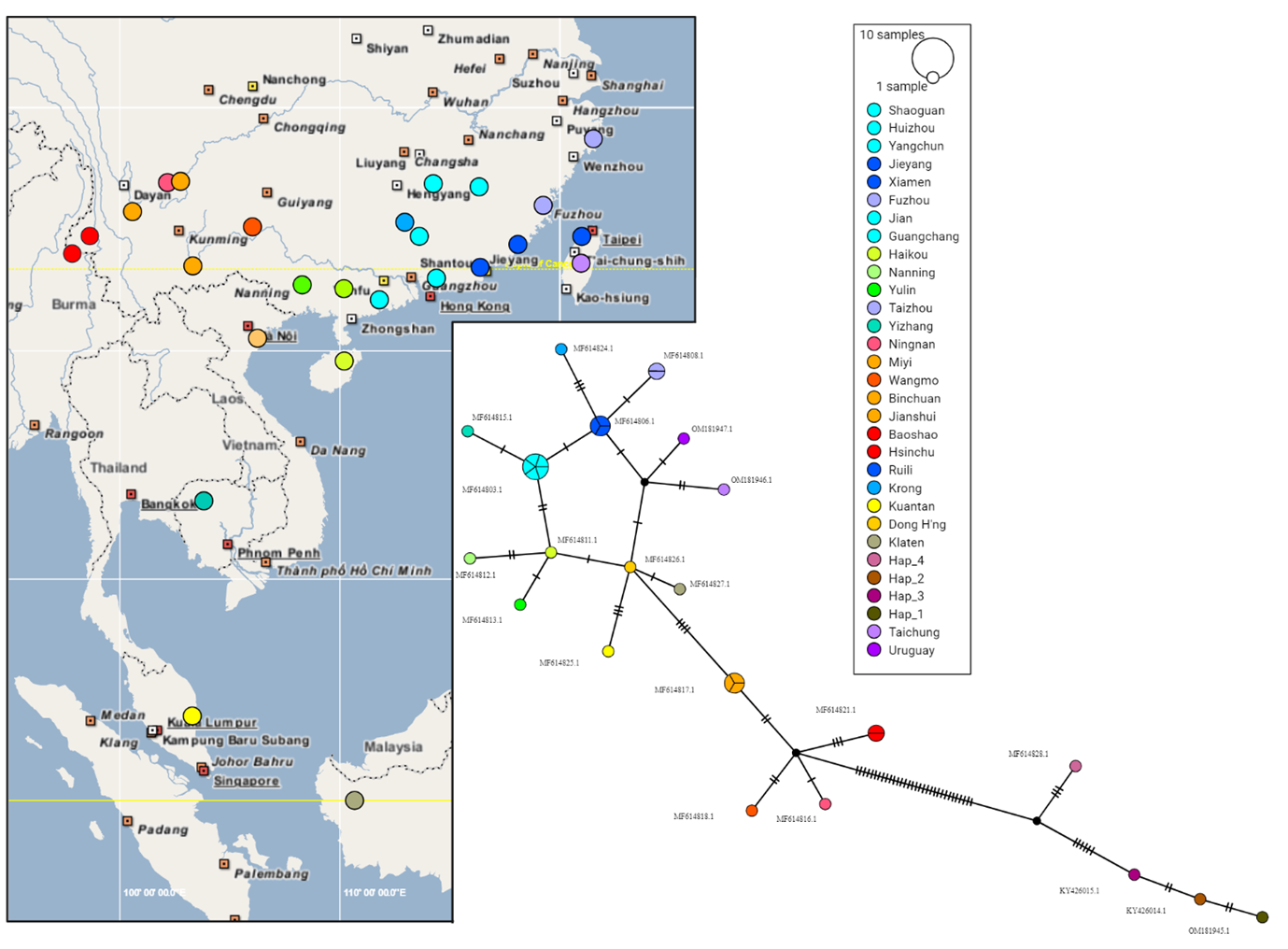

Supplement: Supplementary file 3 — Supplementary Material 3. [file 12864_2026_12811_MOESM3_ESM.tiff]

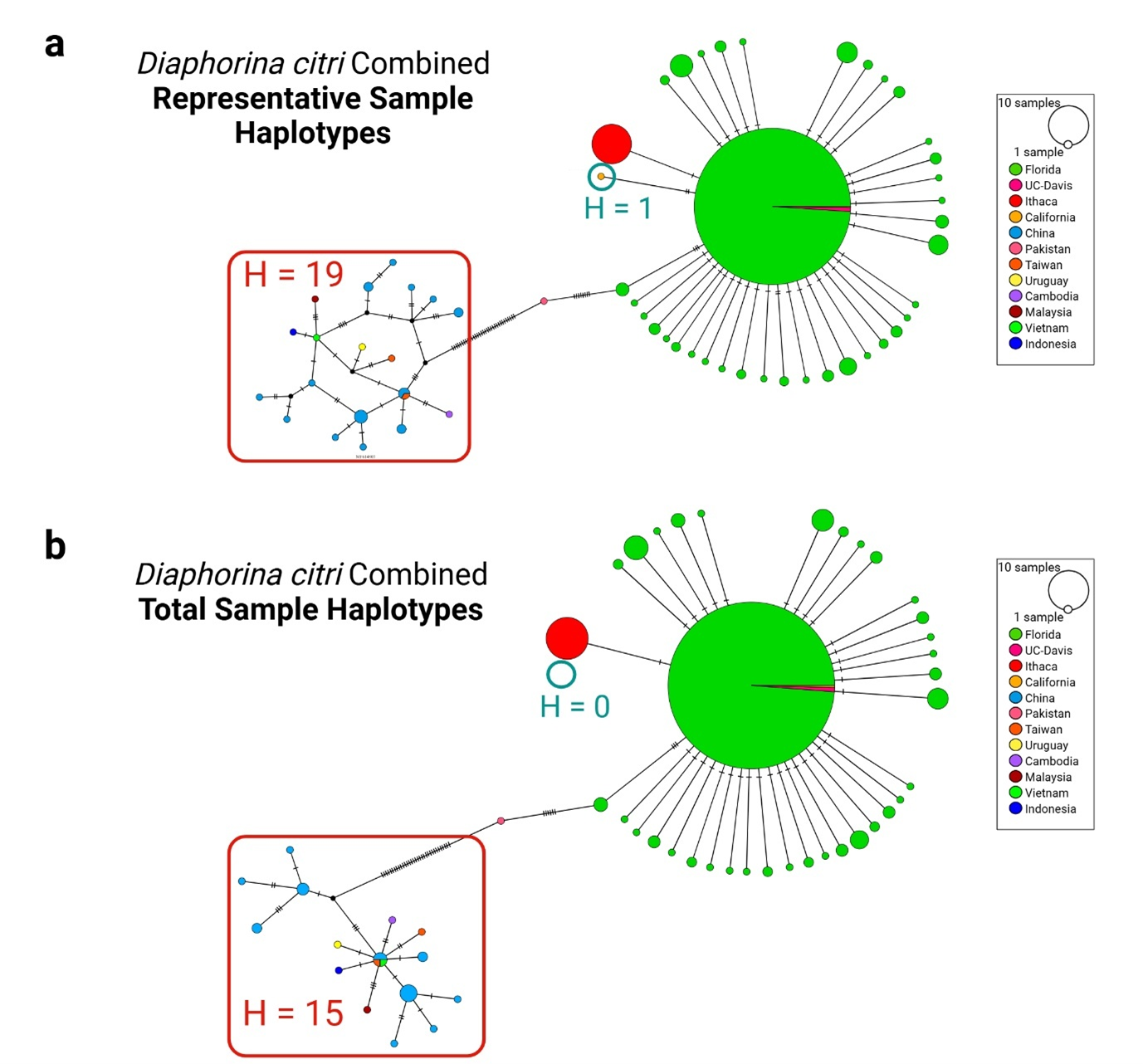

Supplement: Supplementary file 4 — Supplementary Material 4. [file 12864_2026_12811_MOESM4_ESM.tiff]

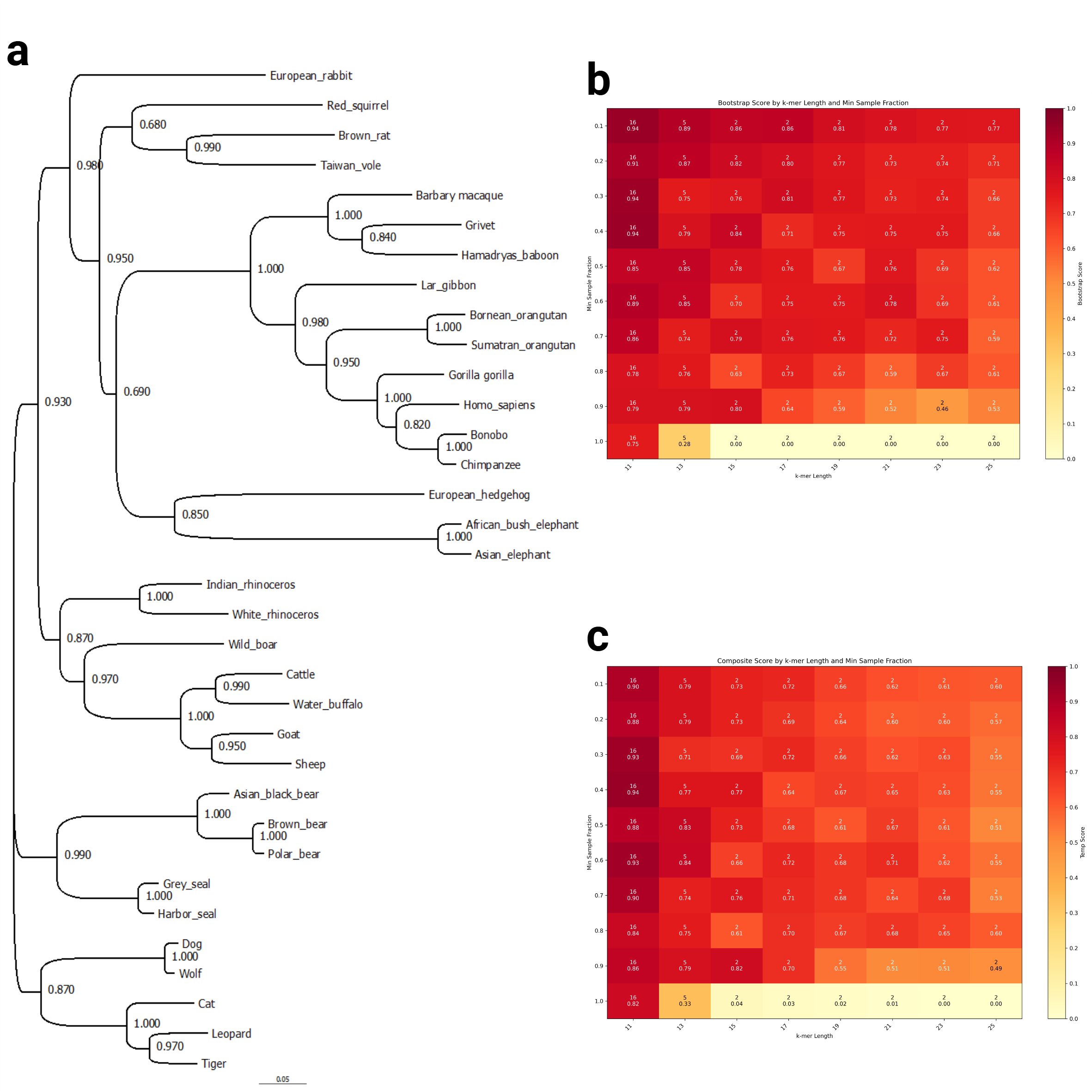

Supplement: Supplementary file 5 — Supplementary Material 5. [file 12864_2026_12811_MOESM5_ESM.tiff]

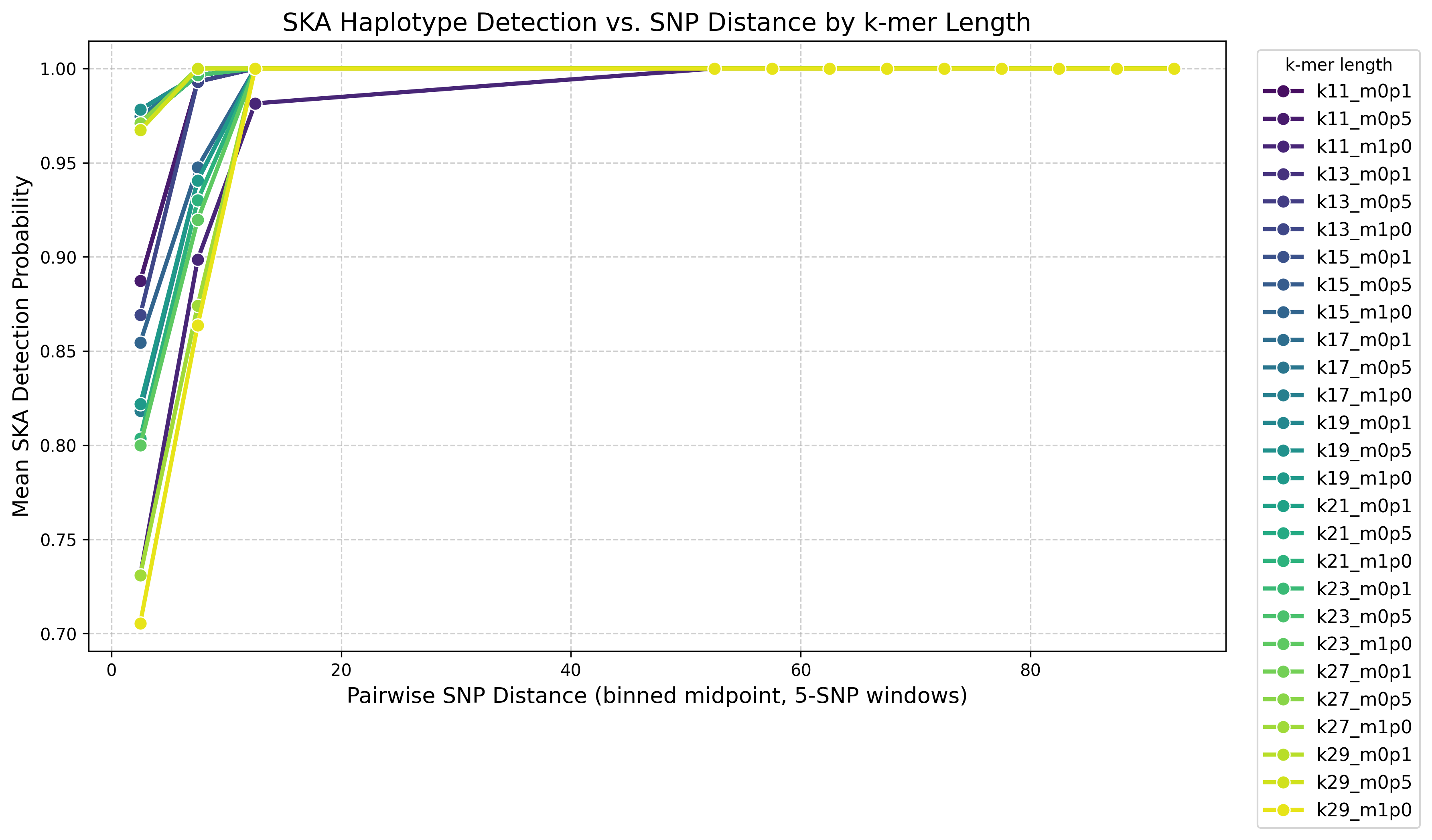

Supplement: Supplementary file 10 — Supplementary Material 10. [file 12864_2026_12811_MOESM10_ESM.tiff]
