# Supplementary material for: Rapid and reproducible haplotyping of complete mitochondrial genomes using split k-mers
Source: BMC Genomics. 2026 Apr 11;27:477. doi: 10.1186/s12864-026-12811-x (PMC13181888; doi:10.1186/s12864-026-12811-x)
Supplement: Supplementary file 7 — Supplementary Material 7. [file 12864_2026_12811_MOESM7_ESM.docx]

**Supplementary data**


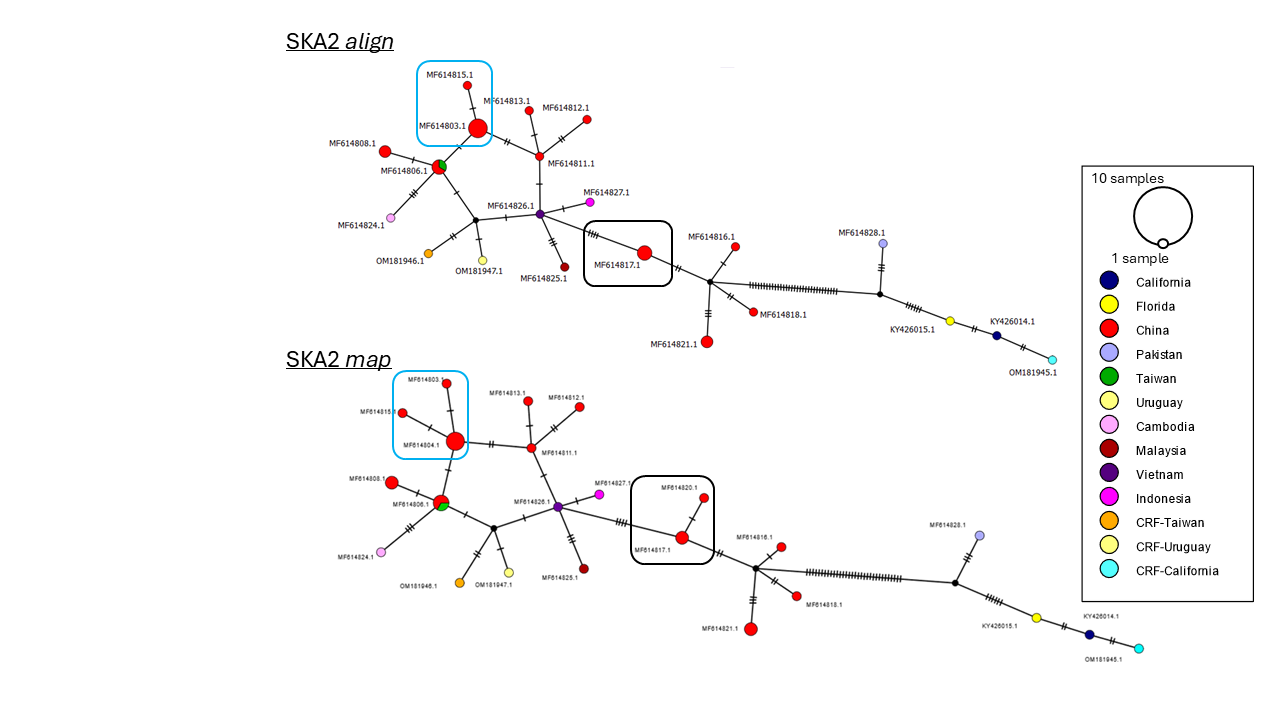


**Figure S1.** TCS Haplotype networks of *D. citri* Genbank dataset produced with SKA *align* and SKA *map* algorithms with *k* = 15 and *m =* 0.9, which was the highest scoring parameter set without repeat masking. The *map* algorithm identified two more haplotypes in the dataset (blue and black boxes) showing that the ska *map* function is more sensitive to discern mtDNA haplotypes. The SNP detected with the *map* function separating MF614803.1 and MF614804.1 is 6744A>T in the NADH dehydrogenase subunit 5 gene. The SNP detected with the *map* function separating MF614820.1 and MF614817.1 is 6746T>C in the NADH dehydrogenase subunit 5 gene.


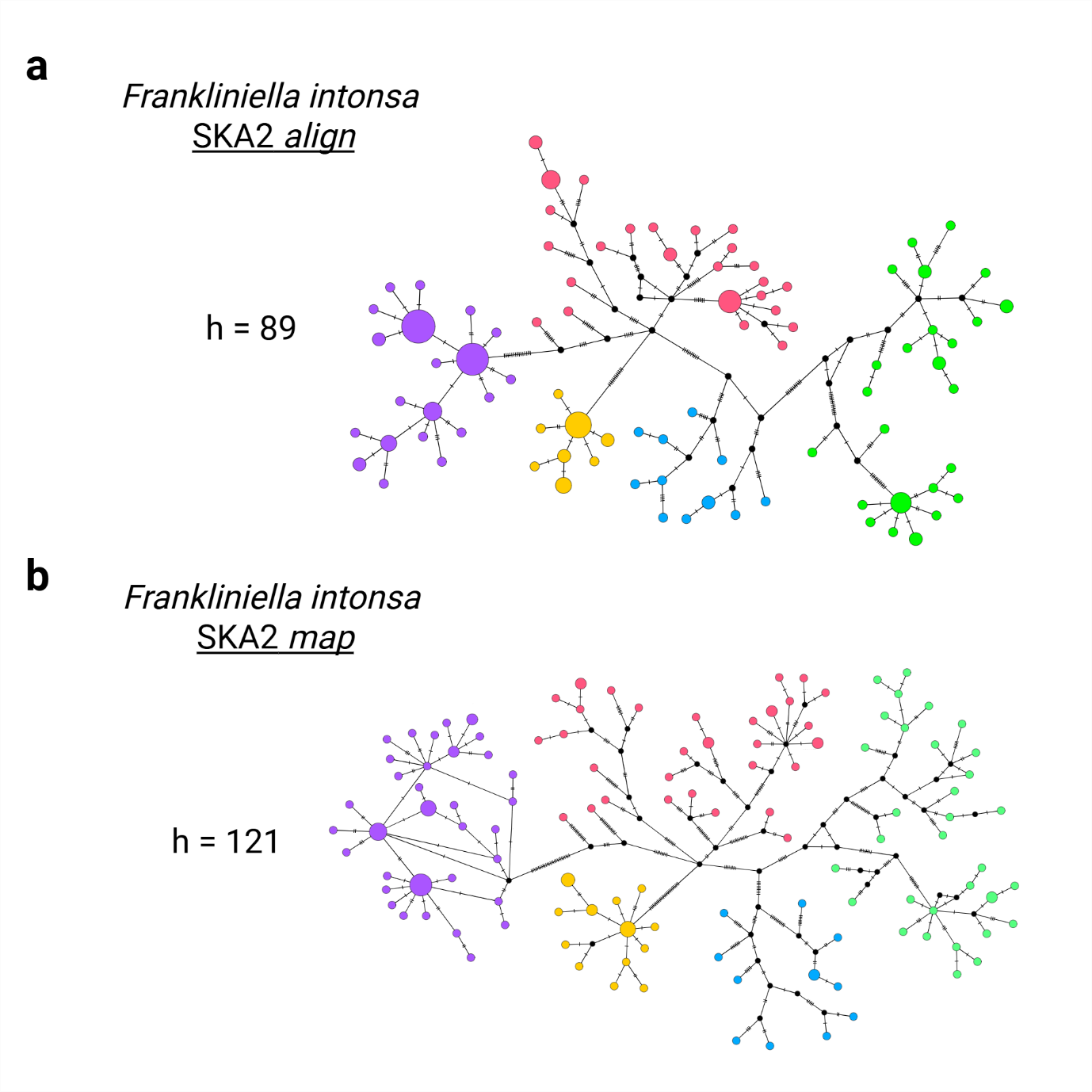


**Figure S2.** Median joining haplotype networks of the *F. intonsa* dataset produced with SKA *align* and SKA *map* algorithms with *k* = 19 and *m =* 0.2. The *map* algorithm identified thirty-two more haplotypes in the dataset showing that the ska *map* function is more sensitive in discerning mtDNA haplotypes.

**
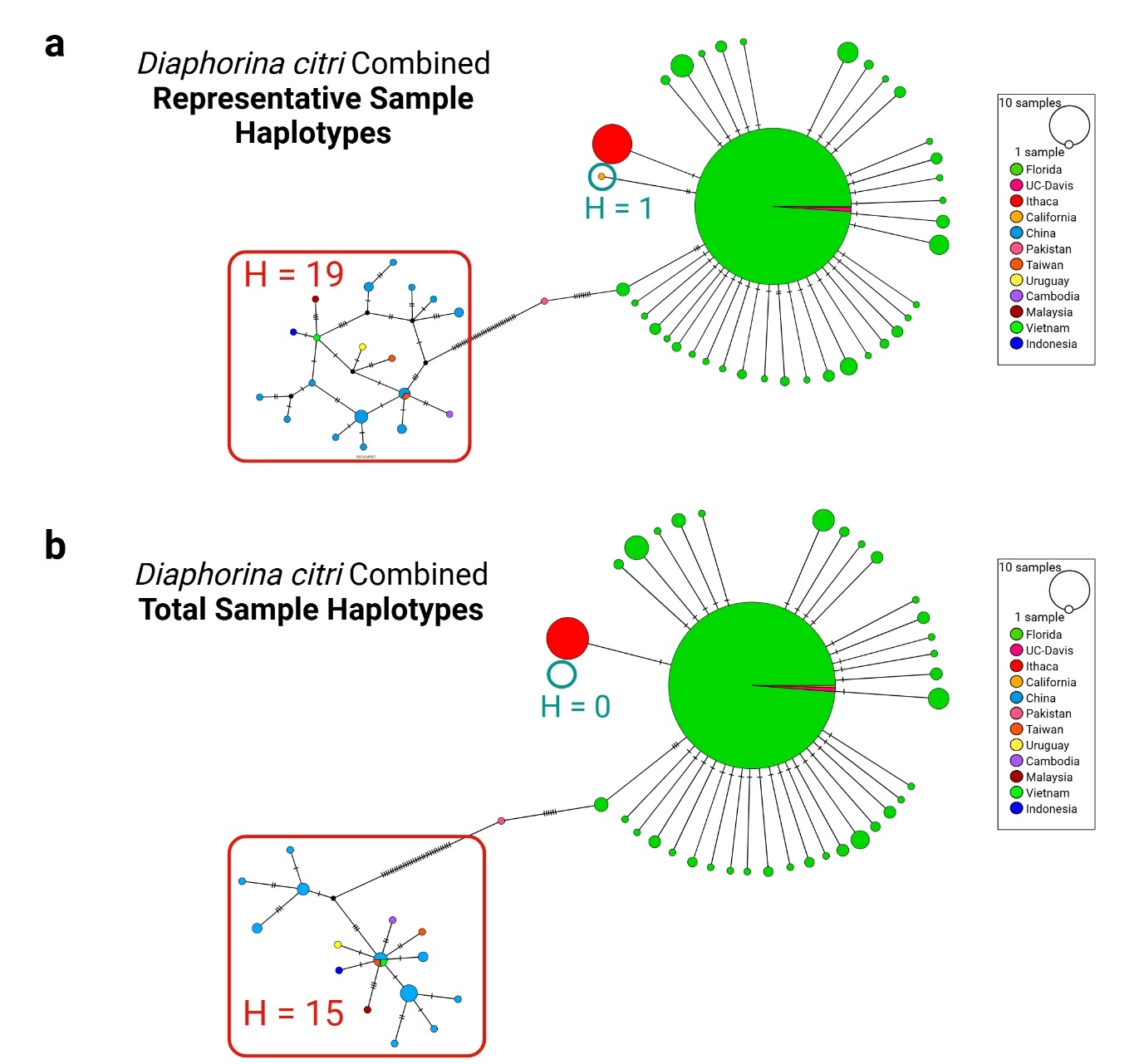
**

**Figure S3.** Two TCS haplotype networks of the *Diaphorina citri* Combined dataset using *k* = 15 and *m* = 90. The top network (a) was made using population assess representative haplotypes and the bottom network (b) used all samples to haplotype. Due to the overabundance of samples from the Florida population, split *k*-mers with SNPs from Asian samples and a California sample were filtered out by the minimum sample fraction filter *m*. This resulted in a lack of haplotype detection and resolution, where four haplotypes of Asian samples were not detected without population assessment and a one-sample haplotype from California was not detected without initial population assessments. After haplotyping the overabundant Florida samples separately using ska *map*, one sample representing each haplotype from the Florida population were refiltered and haplotyped with the rest of the Combined dataset to reduce the impact of population overrepresentation. The *k*-mers unique to a population, containing real SNPs, will not be filtered out as long as the number of samples from that population with the unique *k*-mer/s represent a percentage of the total samples greater than or equal to the minimum sample fraction assigned to *m*.


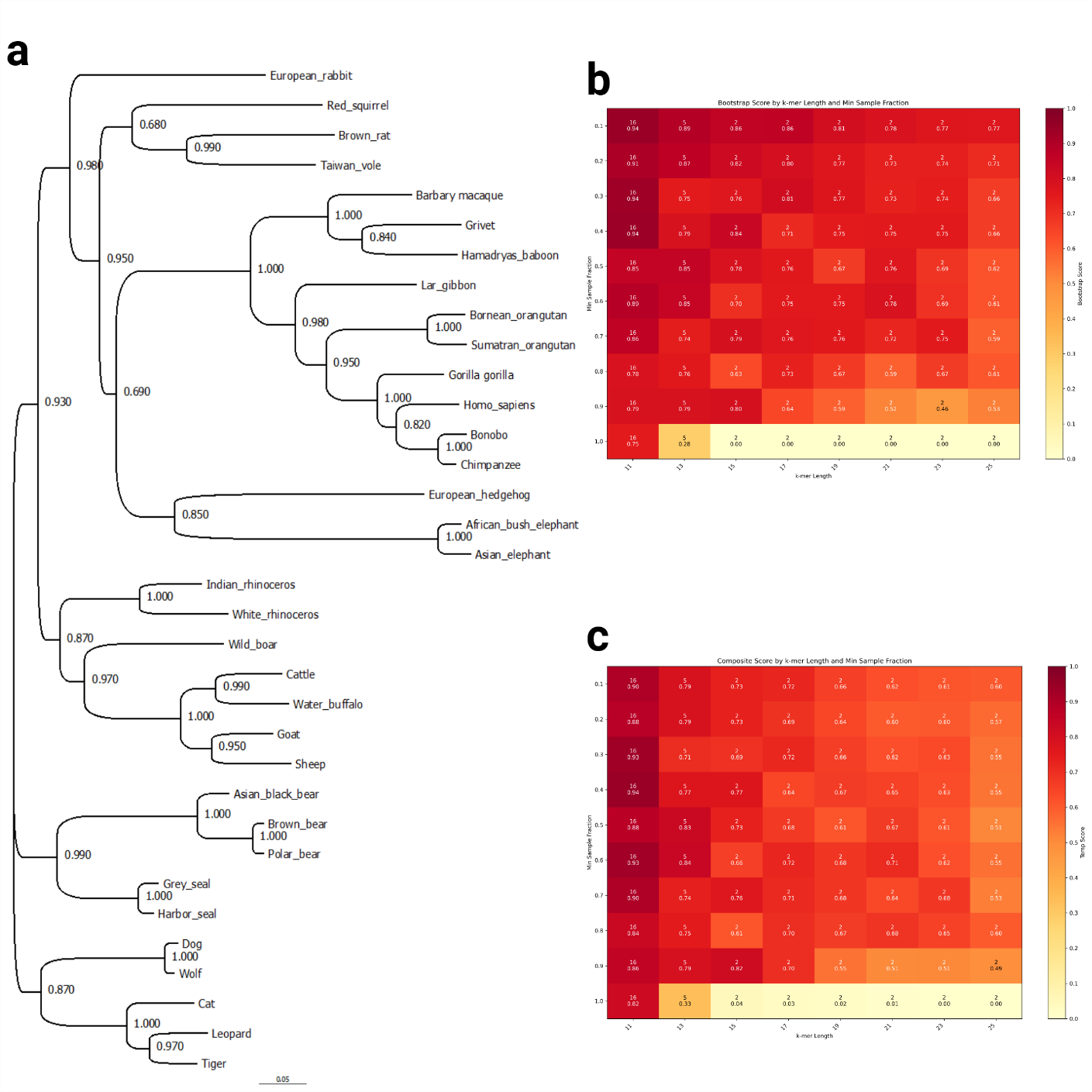


**Figure S4. a.** FastTree phylogenetic tree of 34 mammalian species made with *k* 11 and *m* 40. Tree was built using the optimize-ska-mtDNA.py script where the generalized time-reversible (GTR) model is used. **b.** Heatmap colored by bootstrap support comparing k-mer length and minimum sample fractions. Numbers in each cell are the number of different haplotypes detected in the dataset for each parameter set. **c.** Heatmap of the same structure colored by composite score.


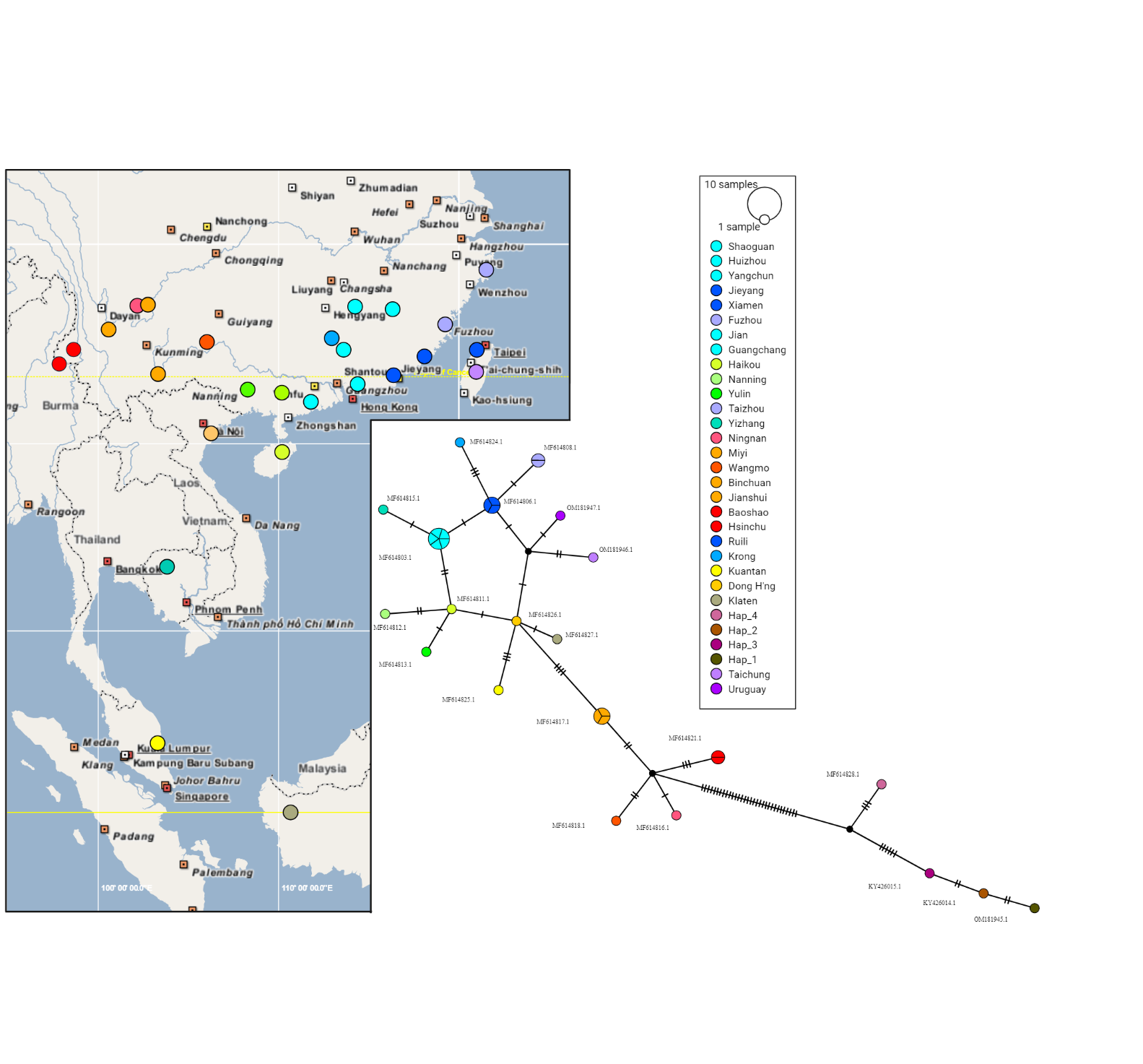


**Figure S5.** TCS haplotype network and map of *D. citri* haplotypes from Southeast Asia produced with the reference free *align* command and dataset from Carlson et al.^16^ Haplotypes identified in the TCS network correlate to regional populations of *D. citri* discerning related but distinct haplotypes in the same or different regions.

**
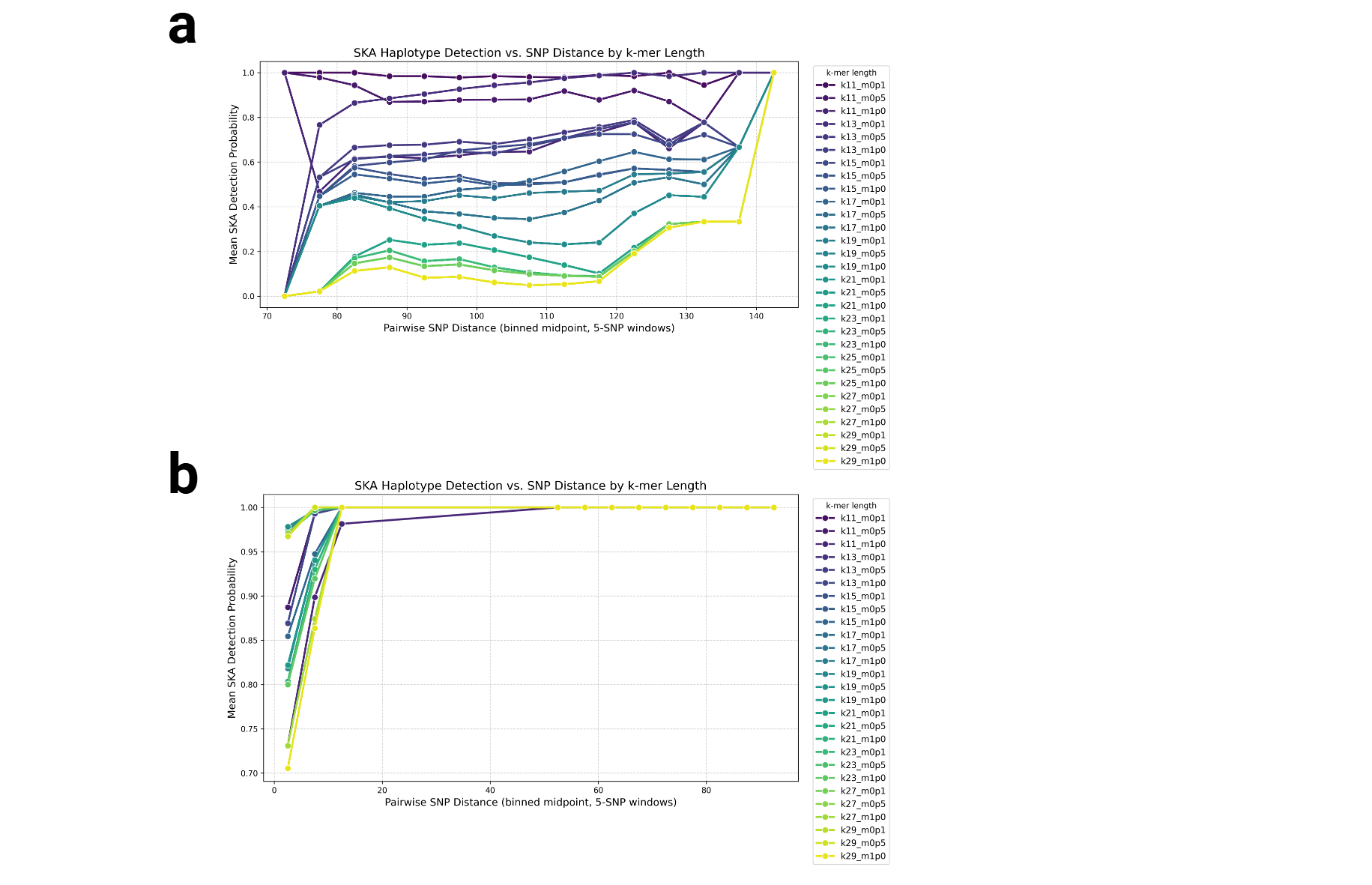
Figure S6. Effect of pairwise SNP distance on SKA haplotype detection across k-mer lengths for lineage-based scenario 4.** Pairwise comparisons between simulated mitochondrial haplotypes were grouped into 5-SNP distance bins, and the mean SKA detection probability was calculated for each bin. Detection probability represents the proportion of haplotype pairs that were assigned to different SKA haplotypes (Detected_By_SKA = 1) within each distance class. Lines show the mean detection probability across distance bins for each tested k-mer length. The x-axis indicates the midpoint of each SNP-distance bin and the y-axis shows the mean probability that SKA distinguished haplotypes at that divergence level. Data is shown for two simulation datasets (2 and 4) with SNP clusters added to test their impact on haplotype detection.

| *Ovis spp.* samples and haplotypes | | |
| --- | --- | --- |
| **Haplotype No.** | **Sample Accessions** | **SNP Distances^1^** |
| H1 | JN181255.1 | 393.83 |
| H2 | HM236188.1 | 328.06 |
| H3 | HM236189.1 | 279.16 |
| H4 | HM236183.1 | 172.67 |
| H5 | HM236180.1, HM236181.1 | 171.47 |
| H6 | KF312238.2 | 170.70 |
| H7 | HM236179.1 | 166.77 |
| H8 | HM236182.1 | 166.14 |
| H9 | MG489885.1 | 163.72 |
| H10 | HM236178.1 | 163.55 |
| H11 | HM236174.1 | 158.70 |
| H12 | HM236175.1 | 153.86 |
| H13 | HM236184.1, KF938360.1 | 148.91 |
| H14 | HM236176.1, HM236177.1 | 148.56 |

**Table S1.** *Ovis spp.* samples and haplotypes as detected by SKA2 *map. ^1^*SNP Distances represent the number of SNPs for that sample found between all samples by pairwise comparisons.

| *Frankliniella intonsa* samples and haplotypes | | |
| --- | --- | --- |
| **Haplotype No.** | **Sample Accessions** | **SNP Distances^1^** |
| H1 | OP546405.1 | 69.57 |
| H2 | OP546494.1 | 68.39 |
| H3 | OP546393.1 | 67.92 |
| H4 | OP546495.1 | 67.83 |
| H5 | OP546399.1 | 67.54 |
| H6 | OP546436.1 | 67.16 |
| H7 | OP546445.1 | 67.16 |
| H8 | OP546492.1 | 66.89 |
| H9 | OP546372.1 | 66.72 |
| H10 | OP546437.1 | 66.69 |
| H11 | OP546368.1 | 66.65 |
| H12 | OP546371.1 | 66.27 |
| H13 | OP546383.1 | 66.22 |
| H14 | OP546395.1 | 66.12 |
| H15 | OP546488.1 | 65.87 |
| H16 | OP546392.1,OP546402.1 | 65.74 |
| H17 | OP546380.1 | 65.69 |
| H18 | OP546459.1 | 65.18 |
| H19 | OP546511.1 | 64.84 |
| H20 | OP546448.1 | 64.83 |
| H21 | OP546431.1 | 64.78 |
| H22 | OP546421.1 | 64.78 |
| H23 | OP546394.1 | 64.67 |
| H24 | OP546463.1 | 64.63 |
| H25 | OP546512.1,OP546513.1,OP546514.1 | 64.11 |
| H26 | OP546508.1,OP546396.1 | 64.09 |
| H27 | OP546477.1 | 64.05 |
| H28 | OP546374.1 | 64.02 |
| H29 | OP546479.1 | 63.95 |
| H30 | OP546433.1 | 63.77 |
| H31 | OP546468.1 | 63.28 |
| H32 | OP546446.1 | 63.05 |
| H33 | OP546452.1 | 62.95 |
| H34 | OP546455.1 | 62.85 |
| H35 | OP546470.1 | 62.83 |
| H36 | OP546466.1 | 62.83 |
| H37 | OP546375.1 | 62.74 |
| H38 | OP546498.1 | 62.66 |
| H39 | OP546515.1 | 62.6 |
| H40 | OP546516.1 | 62.49 |
| H41 | OP546420.1 | 62.38 |
| H42 | OP546500.1 | 62.3 |
| H43 | OP546475.1 | 62.2 |
| H44 | OP546471.1 | 62.18 |
| H45 | OP546442.1 | 62.04 |
| H46 | OP546460.1 | 61.72 |
| H47 | OP546462.1 | 61.37 |
| H48 | OP546478.1 | 61.25 |
| H49 | OP546486.1 | 60.92 |
| H50 | OP546429.1,OP546427.1 | 60.91 |
| H51 | OP546472.1 | 60.9 |
| H52 | OP546482.1 | 60.83 |
| H53 | OP546476.1 | 60.75 |
| H54 | OP546458.1 | 60.66 |
| H55 | OP546501.1 | 60.59 |
| H56 | OP546474.1,OP546376.1,OP546453.1,OP546481.1 | 60.37 |
| H57 | OP546398.1 | 58.35 |
| H58 | OP546435.1 | 58.14 |
| H59 | OP546469.1 | 57.67 |
| H60 | OP546381.1 | 57.38 |
| H61 | OP546401.1 | 57.35 |
| H62 | OP546424.1,OP546423.1 | 57.05 |
| H63 | OP546507.1 | 56.96 |
| H64 | OP546373.1 | 56.91 |
| H65 | OP546480.1 | 56.9 |
| H66 | OP546430.1,OP546426.1 | 56.69 |
| H67 | OP546497.1 | 56.56 |
| H68 | OP546408.1 | 56.5 |
| H69 | OP546415.1 | 56.48 |
| H70 | OP546391.1 | 56.44 |
| H71 | OP546499.1 | 56.19 |
| H72 | OP546422.1 | 56.14 |
| H73 | OP546441.1 | 56.12 |
| H74 | OP546510.1 | 56.11 |
| H75 | OP546443.1,OP546485.1 | 56.02 |
| H76 | OP546509.1 | 56.02 |
| H77 | OP546447.1 | 55.88 |
| H78 | OP546406.1 | 55.85 |
| H79 | OP546491.1 | 55.84 |
| H80 | OP546418.1 | 55.78 |
| H81 | OP546384.1 | 55.69 |
| H82 | OP546465.1 | 55.67 |
| H83 | OP546490.1 | 55.49 |
| H84 | OP546411.1 | 55.4 |
| H85 | OP546417.1 | 55.34 |
| H86 | OP546449.1 | 55.33 |
| H87 | OP546504.1,OP546410.1,OP546432.1,OP546434.1,OP546439.1,OP546440.1,OP546451.1,OP546502.1 | 55.29 |
| H88 | OP546378.1 | 55.28 |
| H89 | OP546400.1 | 55.12 |
| H90 | OP546505.1 | 55.05 |
| H91 | OP546379.1 | 55.02 |
| H92 | OP546416.1 | 54.97 |
| H93 | OP546444.1 | 54.96 |
| H94 | OP546386.1 | 54.94 |
| H95 | OP546454.1 | 54.9 |
| H96 | OP546457.1 | 54.82 |
| H97 | OP546489.1 | 54.58 |
| H98 | OP546390.1 | 54.56 |
| H99 | OP546503.1,OP546409.1,OP546412.1,OP546487.1 | 54.53 |
| H100 | OP546385.1 | 54.5 |
| H101 | OP546388.1 | 54.41 |
| H102 | OP546407.1,OP546403.1 | 54.31 |
| H103 | OP546370.1 | 54.28 |
| H104 | OP546382.1 | 54.21 |
| H105 | OP546450.1 | 54.2 |
| H106 | OP546404.1 | 53.91 |
| H107 | OP546413.1 | 53.91 |
| H108 | OP546461.1 | 53.83 |
| H109 | OP546419.1 | 53.51 |
| H110 | OP546387.1,OP546369.1,OP546389.1,OP546438.1,OP546506.1 | 53.49 |
| H111 | OP546483.1 | 53.39 |
| H112 | OP546414.1 | 53.35 |
| H113 | OP546467.1 | 53.08 |
| H114 | OP546464.1,OP546428.1 | 53.05 |
| H115 | OP546397.1 | 53.05 |
| H116 | OP546493.1 | 53.02 |
| H117 | OP546425.1 | 52.82 |
| H118 | OP546496.1 | 52.4 |
| H119 | OP546484.1 | 52.39 |
| H120 | OP546473.1,OP546377.1 | 52.02 |
| H121 | OP546456.1 | 51.82 |

**Table S2.** *Frankliniella intonsa* samples and haplotypes as detected by SKA2 *map. ^1^*SNP Distances represent the number of SNPs for that sample found between all samples by pairwise comparisons.

| *Diaphorina citri* Genbank samples and haplotypes | | |
| --- | --- | --- |
| **Haplotype No.** | **Sample Accessions** | **SNP Distances^1^** |
| H1 | OM181945.1 | 46.43 |
| H2 | KY426014.1 | 45.47 |
| H3 | KY426015.1 | 45.35 |
| H4 | MF614828.1 | 37.48 |
| H5 | MF614821.1, MF614822.1 | 14.38 |
| H6 | MF614824.1 | 13.05 |
| H7 | MF614818.1 | 12.62 |
| H8 | MF614825.1 | 12.6 |
| H9 | MF614812.1 | 12.07 |
| H10 | MF614816.1 | 12.02 |
| H11 | OM181946.1 | 11.5 |
| H12 | MF614817.1, MF614819.1, MF614820.1 | 11.17 |
| H13 | MF614813.1 | 11.1 |
| H14 | MF614815.1 | 10.98 |
| H15 | MF614827.1 | 10.67 |
| H16 | MF614808.1, MF614814.1 | 10.48 |
| H17 | OM181947.1 | 10.23 |
| H18 | MF614811.1 | 10.13 |
| H19 | MF614803.1,MF614804.1, MF614805.1, MF614809.1, MF614810.1 | 10.02 |
| H20 | MF614826.1 | 9.7 |
| H21 | MF614806.1, MF614807.1, MF614823.1 | 9.58 |

**Table S3.** *Diaphorina citri* Genbank samples and haplotypes as detected by SKA2 *map. ^1^*SNP Distances represent the number of SNPs for that sample found between all samples by pairwise comparisons.

| *Diaphorina citri* Combined samples and haplotypes | | |
| --- | --- | --- |
| **Haplotype No.** | **Sample Accessions** | **SNP Distances^1^** |
| H1 | MF614822.1,MF614821.1 | 37.37 |
| H2 | MF614825.1 | 37.19 |
| H3 | MF614815.1 | 37.02 |
| H4 | MF614812.1 | 36.95 |
| H5 | MF614824.1 | 36.95 |
| H6 | MF614803.1,MF614804.1,MF614805.1,MF614809.1,MF614810.1 | 36.89 |
| H7 | OM181946.1 | 36.7 |
| H8 | MF614814.1,MF614808.1 | 36.7 |
| H9 | MF614813.1 | 35.98 |
| H10 | MF614818.1 | 35.94 |
| H11 | MF614826.1 | 35.77 |
| H12 | OM181947.1 | 35.72 |
| H13 | MF614816.1 | 35.7 |
| H14 | MF614827.1 | 35.22 |
| H15 | MF614811.1 | 35 |
| H16 | MF614806.1,MF614807.1,MF614823.1 | 34.23 |
| H17 | MF614819.1,MF614820.1,MF614817.1 | 33.69 |
| H18 | MF614828.1 | 24.54 |
| H19 | CA-G3 | 23.64 |
| H20 | 29,ACP29 | 23.35 |
| H21 | AB-A12,AC-C3,AC-D2,AD-A11,AD-A12,AD-B7,AD-C7 | 22.92 |
| H22 | 14,4,5,BA-B4 | 22.44 |
| H23 | 38,ACP38 | 22.44 |
| H24 | 65,CA-B8,CA-C7,CA-D9,CA-E5,CA-F2,CA-F5,CA-F9,CA-H2,CA-H7,CA-H8,CA-H9 | 22.44 |
| H25 | AA-A1,AA-F4 | 22.44 |
| H26 | AA-B6 | 22.44 |
| H27 | AA-D4 | 22.44 |
| H28 | AA-F11,AB-F1 | 22.44 |
| H29 | AA-F9 | 22.44 |
| H30 | AB-A8 | 22.44 |
| H31 | AB-B9 | 22.44 |
| H32 | AC-C12 | 22.44 |
| H33 | AC-E3 | 22.44 |
| H34 | AC-F3 | 22.44 |
| H35 | AC-G5 | 22.44 |
| H36 | AD-B4 | 22.44 |
| H37 | AD-B9,AD-E5,AD-F3 | 22.44 |
| H38 | B27 | 22.44 |
| H39 | BA-A11,BA-B11,BA-C8,BA-D10,BA-D7,BA-E8,BA-F11,BA-F8,BA-G10 | 22.44 |
| H40 | BA-B2 | 22.44 |
| H41 | CA-D4 | 22.44 |
| H42 | D12,D21,D22 | 22.44 |
| H43 | DA-G3 | 22.44 |
| H44 | DB-C1,DB-D4,DB-E1,DB-E7,DB-E8,DB-F2,DB-F4,DB-G7,DB-G9,DB-H7 | 22.44 |
| H45 | OM181945.1 | 22.44 |
| H46 | AC-C5,AC-H4,AD-F11 | 22.43 |
| H47 | 19E,20E,22E,24E,2E,3E,4E,7E,9E,A5,E5A,E5B,E5E,E6,L2,SE14,SE15,SE18,SE19,SE20,SE4,SE6,SE8,T11,T12,T13,T14,T15,T16,T3,T4,T5,T6,T9,V1,V12,V2 | 22.41 |
| H48 | AA-C9 | 22.38 |
| H49 | 28,AC-B3,ACP28 | 22.36 |
| H50 | 96,DA-F4 | 22.36 |
| H51 | DA-C2,DA-D1,DA-H3 | 22.36 |
| H52 | BA-A1,BA-F1,BA-H2 | 22.31 |
| H53 | AB-C8,AB-G5,B14,B21 | 22.08 |
| H54 | BA-G4 | 21.99 |
| H55 | CA-C10,CA-D10 | 21.91 |
| H56 | 13,16,18,2,20,21,22,26,27,3,33,36,39,40,46,47,49,50,51,52,53,54,55,56,57,58,59,6,60,61,64,66,68,70,71,72,73,74,76,77,78,79,80,81,82,83,85,86,87,88,89,9,90,91,93,94,95,A21,A23,A24,AA-A12,AA-A2,AA-A4,AA-B1,AA-B11,AA-B12,AA-B2,AA-B4,AA-B7,AA-B9,AA-C1,AA-C12,AA-C4,AA-C5,AA-D12,AA-D8,AA-E1,AA-E12,AA-E9,AA-F1,AA-F12,AA-G11,AA-G12,AA-G5,AA-G6,AA-G7,AA-G9,AA-H1,AA-H11,AA-H3,AA-H4,AA-H7,AA-H8,AA-H9,AB-A10,AB-A11,AB-A3,AB-A5,AB-A6,AB-A7,AB-B1,AB-B10,AB-B12,AB-B4,AB-B5,AB-B6,AB-B7,AB-B8,AB-C10,AB-C5,AB-C6,AB-C9,AB-D10,AB-D12,AB-D2,AB-D5,AB-D6,AB-D8,AB-E1,AB-E10,AB-E11,AB-E2,AB-E7,AB-E9,AB-F10,AB-F11,AB-F12,AB-F3,AB-F5,AB-F9,AB-G1,AB-G12,AB-G6,AB-G8,AB-G9,AB-H10,AB-H2,AB-H5,AB-H6,AB-H7,AB-H9,AC-A10,AC-A11,AC-A12,AC-A3,AC-A4,AC-A5,AC-A6,AC-A7,AC-B1,AC-B11,AC-B2,AC-B4,AC-B6,AC-B7,AC-B8,AC-B9,AC-C11,AC-C4,AC-C8,AC-C9,AC-D1,AC-D11,AC-D3,AC-D4,AC-D5,AC-D7,AC-D8,AC-D9,AC-E10,AC-E11,AC-E12,AC-E2,AC-E4,AC-E5,AC-E6,AC-E7,AC-E8,AC-F4,AC-F5,AC-F8,AC-G1,AC-G10,AC-G11,AC-G12,AC-G3,AC-G9,AC-H1,AC-H2,AC-H3,AC-H6,AC-H8,AC-H9,ACP25,ACP26,ACP27,ACP33,ACP36,ACP39,ACP40,ACP46,ACP47,ACPA18,ACPA21,ACPA23,ACPA24,AD-A1,AD-A2,AD-A6,AD-B1,AD-B12,AD-B2,AD-B3,AD-B5,AD-B6,AD-C1,AD-C2,AD-C3,AD-C4,AD-C5,AD-C6,AD-C8,AD-D10,AD-D12,AD-D5,AD-D6,AD-D7,AD-E10,AD-E12,AD-E4,AD-E9,AD-F10,AD-F12,AD-F2,AD-F4,AD-G1,AD-G11,AD-G2,AD-G3,AD-G4,AD-G5,AD-G8,B1,B12,B13,B24,B3,B7,BA-A10,BA-A12,BA-A4,BA-A8,BA-A9,BA-B10,BA-B3,BA-B5,BA-B8,BA-B9,BA-C10,BA-C11,BA-C12,BA-C4,BA-C5,BA-C6,BA-C9,BA-D12,BA-D6,BA-D8,BA-D9,BA-E10,BA-E11,BA-E12,BA-E2,BA-E4,BA-E5,BA-E7,BA-E9,BA-F10,BA-F12,BA-F5,BA-F6,BA-F9,BA-G12,BA-G2,BA-G3,BA-G9,BA-H1,BA-H3,BA-H4,BA-H5,BA-H8,BA-H9,C1,C10,C11,C17,C21,C22,C23,C24,C3,C7,C8,CA-A1,CA-A10,CA-A11,CA-A12,CA-A3,CA-A4,CA-A5,CA-A6,CA-A7,CA-A9,CA-B1,CA-B11,CA-B12,CA-B2,CA-B3,CA-B4,CA-B5,CA-B6,CA-B7,CA-B9,CA-C1,CA-C11,CA-C12,CA-C2,CA-C3,CA-C4,CA-C5,CA-C8,CA-D1,CA-D11,CA-D12,CA-D2,CA-D3,CA-D6,CA-D7,CA-D8,CA-E1,CA-E10,CA-E11,CA-E12,CA-E3,CA-E4,CA-E7,CA-E8,CA-F1,CA-F3,CA-F4,CA-G1,CA-G10,CA-G11,CA-G12,CA-G2,CA-G4,CA-G5,CA-G6,CA-G7,CA-G9,CA-H1,CA-H10,CA-H11,CA-H3,CA-H4,CA-H5,CA-H6,D14,D23,D24,D6,D9,DA-A1,DA-A10,DA-A11,DA-A12,DA-A3,DA-A4,DA-A5,DA-A6,DA-A7,DA-A8,DA-A9,DA-B1,DA-B10,DA-B11,DA-B2,DA-B3,DA-B5,DA-B6,DA-B7,DA-B8,DA-B9,DA-C1,DA-C10,DA-C11,DA-C12,DA-C3,DA-C4,DA-C5,DA-C6,DA-C9,DA-D10,DA-D12,DA-D2,DA-D3,DA-D4,DA-D5,DA-D6,DA-D7,DA-D8,DA-E1,DA-E10,DA-E11,DA-E12,DA-E2,DA-E4,DA-E7,DA-E8,DA-E9,DA-F10,DA-F11,DA-F12,DA-F2,DA-F3,DA-F5,DA-F6,DA-F7,DA-F8,DA-F9,DA-G1,DA-G10,DA-G11,DA-G12,DA-G2,DA-G5,DA-G6,DA-G8,DA-G9,DA-H1,DA-H10,DA-H11,DA-H2,DA-H4,DA-H5,DA-H6,DA-H8,DA-H9,DB-A1,DB-A10,DB-A11,DB-A12,DB-A2,DB-A3,DB-A5,DB-A6,DB-A8,DB-A9,DB-B1,DB-B11,DB-B12,DB-B2,DB-B3,DB-B4,DB-B5,DB-B6,DB-B7,DB-B8,DB-B9,DB-C10,DB-C11,DB-C12,DB-C2,DB-C3,DB-C5,DB-C6,DB-D1,DB-D10,DB-D12,DB-D2,DB-D3,DB-D5,DB-D9,DB-E10,DB-E11,DB-E2,DB-E3,DB-E4,DB-E6,DB-E9,DB-F10,DB-F11,DB-F12,DB-F3,DB-F5,DB-F6,DB-F7,DB-F8,DB-G1,DB-G10,DB-G11,DB-G2,DB-G3,DB-G4,DB-G5,DB-G6,DB-G8,DB-H10,DB-H12,DB-H2,DB-H4,DB-H5,DB-H6,DB-H8,DB-H9,H1,H2,H3,I3,I9,KY426014.1,KY426015.1 | 21.45 |

**Table S4.** *Diaphorina citri* Combined samples and haplotypes as detected by SKA2 *map. ^1^*SNP Distances represent the number of SNPs for that sample found between all samples by pairwise comparisons.

| **^1^Hap1** | **^2^Hap2** | **^3^n_Hap1** | **^4^n_Hap2** | **^5^Delta (δ)** | **^6^*p*_wilcox** | **^7^*p*_adj_BH** | **^8^sig** |
| --- | --- | --- | --- | --- | --- | --- | --- |
| H39 | H56 | 9 | 412 | -0.88835 | 5.00E-06 | 0.00014 | *** |
| H44 | H56 | 10 | 412 | 0.803884 | 1.38E-05 | 0.000193 | *** |
| H24 | H39 | 11 | 9 | 1 | 9.50E-05 | 0.000857 | *** |
| H39 | H44 | 9 | 10 | -1 | 1.22E-04 | 0.000857 | *** |
| H24 | H44 | 11 | 10 | -0.85455 | 1.06E-03 | 0.004992 | ** |
| H21 | H44 | 7 | 10 | -0.97143 | 1.08E-03 | 0.004992 | ** |
| H21 | H39 | 7 | 9 | 0.857143 | 1.27E-03 | 0.004992 | ** |
| H39 | H46 | 9 | 3 | -1 | 1.60E-03 | 0.004992 | ** |
| H39 | H51 | 9 | 3 | -1 | 1.60E-03 | 0.004992 | ** |
| H24 | H52 | 11 | 3 | 1 | 1.26E-02 | 0.035374 | * |
| H44 | H52 | 10 | 3 | 1 | 1.41E-02 | 0.035929 | * |
| H51 | H56 | 3 | 412 | 0.796117 | 1.75E-02 | 0.040734 | * |
| H52 | H56 | 3 | 412 | -0.78641 | 1.89E-02 | 0.040734 | * |
| H21 | H51 | 7 | 3 | -1 | 2.27E-02 | 0.045309 | * |
| H24 | H51 | 11 | 3 | -0.87879 | 2.93E-02 | 0.054642 | ns |
| H21 | H52 | 7 | 3 | 0.809524 | 6.49E-02 | 0.113592 | ns |
| H46 | H52 | 3 | 3 | 1 | 7.65E-02 | 0.119035 | ns |
| H51 | H52 | 3 | 3 | 1 | 7.65E-02 | 0.119035 | ns |
| H21 | H24 | 7 | 11 | -0.48052 | 1.03E-01 | 0.151877 | ns |
| H39 | H52 | 9 | 3 | -0.33333 | 1.24E-01 | 0.173121 | ns |
| H44 | H46 | 10 | 3 | 0.6 | 1.51E-01 | 0.201047 | ns |
| H21 | H56 | 7 | 412 | -0.26907 | 2.22E-01 | 0.282884 | ns |
| H21 | H46 | 7 | 3 | -0.42857 | 3.62E-01 | 0.429527 | ns |
| H46 | H51 | 3 | 3 | -0.55556 | 3.83E-01 | 0.429527 | ns |
| H24 | H56 | 11 | 412 | 0.154016 | 3.84E-01 | 0.429527 | ns |
| H46 | H56 | 3 | 412 | 0.278317 | 4.07E-01 | 0.438373 | ns |
| H44 | H51 | 10 | 3 | 0.333333 | 4.47E-01 | 0.463424 | ns |
| H24 | H46 | 11 | 3 | -0.09091 | 8.76E-01 | 0.87627 | ns |

**Table S5.** Pairwise differences in ln(CLas_abs + 1) titer were evaluated using Wilcoxon rank-sum tests. Effect sizes are reported as ^5^Cliff’s delta (δ) with 95% confidence intervals. The magnitude of δ is interpreted as negligible (< 0.147), small (0.147–0.33), medium (0.33–0.474), or large (> 0.474). The sign of ^5^Delta (δ) indicates direction: positive values indicate higher titers in haplotype 1 (^1^Hap1) relative to haplotype 2 (^2^Hap2), whereas negative values indicate lower titers in haplotype 1 relative to haplotype 2. Sample sizes for each haplotype in each comparison are shown (^3^n_Hap1, ^4^n_Hap2). *P*-values (^7^*p*_adj_BH) were adjusted for multiple comparisons using the Benjamini–Hochberg false discovery rate (FDR) correction. Significance levels (^8^Sig) are indicated as *** (p < 0.001), ** (p < 0.01), * (p < 0.05), and ns (not significant; p ≥ 0.05). Haplotype pairs with small sample sizes (e.g., n = 3) may yield extreme effect size estimates and should be interpreted with caution.

| **Metric** | **Scenario 1** | **Scenario 2** | **Scenario 3** | **Scenario 4** |
| --- | --- | --- | --- | --- |
| **^1^Sequences analyzed** | 144 | 150 | 150 | 150 |
| **^2^Total sites analyzed** | 15,222 | 15,222 | 15,222 | 15,222 |
| **^3^Polymorphic sites (S)** | 4,593 | 5,147 | 10,603 | 623 |
| **^4^Total mutations (Eta)** | 5,163 | 5,833 | 14,998 | 630 |
| **^5^Singleton mutations (Eta(s))** | 1,675 | 4,385 | 12,181 | 156 |
| **^6^Average pairwise differences (k)** | 240.894 | 100.623 | 241.784 | 68.474 |
| **^7^Nucleotide diversity (π)** | 0.01583 | 0.00661 | 0.01588 | 0.00450 |
| **^8^Theta from Eta** | 931.352 | 1044.496 | 2685.641 | 112.812 |
| **^9^Number of haplotypes (h)** | 90 | 123 | 150 | 127 |
| **^10^Haplotype diversity (Hd)** | 0.995 | 0.997 | 1.000 | 0.997 |
| **^11^Haplotypes detected by ska-mtdna** | 90 | 123 | 150 | 104 |

**Table S6.** Summary statistics of mitochondrial sequence diversity and haplotype detection across simulated datasets calculated by DnaSP6. Metrics represent standard population genetic statistics calculated from sequence alignments. ^1^*Sequences analyzed* indicates the number of individual mitochondrial genomes included in the analysis. ^2^*Total sites analyzed* refers to the length of the alignment used for calculations. ^3^*Polymorphic sites (S)* represent the number of nucleotide positions that vary among sequences. ^4^*Total mutations (Eta)* is the total number of mutational events inferred across all sequences. ^5^*Singleton mutations (Eta(s))* are mutations observed in only a single sequence. ^6^*Average pairwise differences (k)* is the mean number of nucleotide differences between all pairwise sequence comparisons. ^7^*Nucleotide diversity (π)* represents the average proportion of nucleotide differences per site between sequences. ^8^*Theta from Eta* is an estimate of the population mutation rate derived from the total number of mutations. ^9^*Number of haplotypes (h)* indicates the count of unique mitochondrial haplotypes present in the dataset. ^10^*Haplotype diversity (Hd)* represents the probability that two randomly selected sequences belong to different haplotypes. ^11^*Haplotypes detected by ska-mtdna* indicates the number of haplotypes recovered by the split k-mer haplotyping pipeline.
